# Persistent Altered Sensorium in a Patient Presenting With Acute Metabolic Encephalopathy From Diabetic Ketoacidosis and Metastatic Recurrent Melanoma of the Lung and Brain

**DOI:** 10.7759/cureus.85023

**Published:** 2025-05-29

**Authors:** Hassan Allahrakha, Francis Fischer, Keshav Poddar

**Affiliations:** 1 Internal Medicine, Methodist Health System, Dallas, USA

**Keywords:** acute metabolic encephalopathy, dka, melanoma, metastatic, metastatic melanoma

## Abstract

Skin cancer, including melanoma, is a common type of cancer. Melanomas can develop anywhere on the skin and frequently metastasize to organs such as the brain, lungs, liver, and bones.

We present the case of a 65-year-old male diagnosed with melanoma metastasis to his brain, causing expressive aphasia. This case is unique because the patient had undergone resection of stage IIC melanoma nine years prior. He initially presented with encephalopathy attributed to diabetic ketoacidosis, but persistent symptoms led to the discovery of brain metastases. He was ultimately diagnosed with metastatic melanoma and underwent whole-brain radiation and a trial of disease-modifying treatment with dabrafenib/trametinib. This case report aims to explore the disease progression of melanoma with metastasis to the brain and to highlight the importance of keeping brain metastasis high on the differential for encephalopathy in patients who have a history of melanoma, regardless of treatment resolution.

## Introduction

Melanomas are malignant tumors that arise from the uncontrolled proliferation of melanocytes in the epidermis. Melanocytes are found primarily in the skin within the junction of the dermis and epidermis. Causes of melanoma include immunological, genetic, and environmental factors. Over the past few decades, the incidence and prevalence of melanoma in the United States have significantly increased. The estimated lifetime risk of developing melanoma is 1 in 74, compared with 1 in 1,500 in 1935 [1]. Approximately 10-20% of melanoma patients have brain metastases at the time of distant metastatic diagnosis, with up to 50% developing them during their lifetime [1]. Clinical evidence indicates that melanoma preferentially metastasizes to organs such as the brain, lungs, and liver. Melanoma often has a protracted disease course, in which patients have a disease-free period following the surgical excision of the primary tumor, only to discover visceral metastases months, years, or even decades later.

Historically, the prognosis for patients with brain metastases has been poor, with overall survival typically less than six months. However, there have been improvements in overall mortality and quality of life in recent years as a result of advanced research, immunotherapy, and advancements in targeted therapies. The introduction of immunotherapy and BRAF/MEK inhibitors has transformed the therapeutic landscape, leading to improved survival rates and offering new hope to patients with advanced disease [2].

Immunotherapy such as anti-PD-1 agents (nivolumab, pembrolizumab) has shown efficacy in melanoma brain metastases, with intracranial response rates of 20-30% [2]. However, due to the patient's BRAF mutation and rapid disease progression, targeted therapy was prioritized.

Individual responses to these treatments can vary widely, and certain metastatic patterns or presentations remain poorly understood or underreported. The management of melanoma depends primarily on the location, depth, and stage of clinical presentation.

## Case presentation

A 65-year-old male with a history of type 2 diabetes, hypertension, hyperlipidemia, and stage IIC melanoma resected in 2013 presented for abdominal pain, nausea, weakness, vomiting, and altered mental status. Per the patient’s wife, he had been experiencing progressive fatigue and weakness in the few days leading up to admission. This was associated with weight loss and poor appetite. Initial lab work was significant for a glucose level of 1,179 mg/dL, pH of 6.9 (normal 7.35-7.45), white blood cell (WBC) count of 41,000 WBCs/µL (normal 4,500 - 11,000 WBCs/µL), anion gap of 23 mEq/L (normal 4-12 mEq/L), and beta-hydroxybutyrate level of 12.8 mmol/L (normal <0.5 mmol/L). The patient was diagnosed with diabetic ketoacidosis (DKA) and treated appropriately with fluids and an insulin drip. His initial altered mental status was thought to be secondary to the metabolic encephalopathy of his DKA episode.

**Figure 1:**
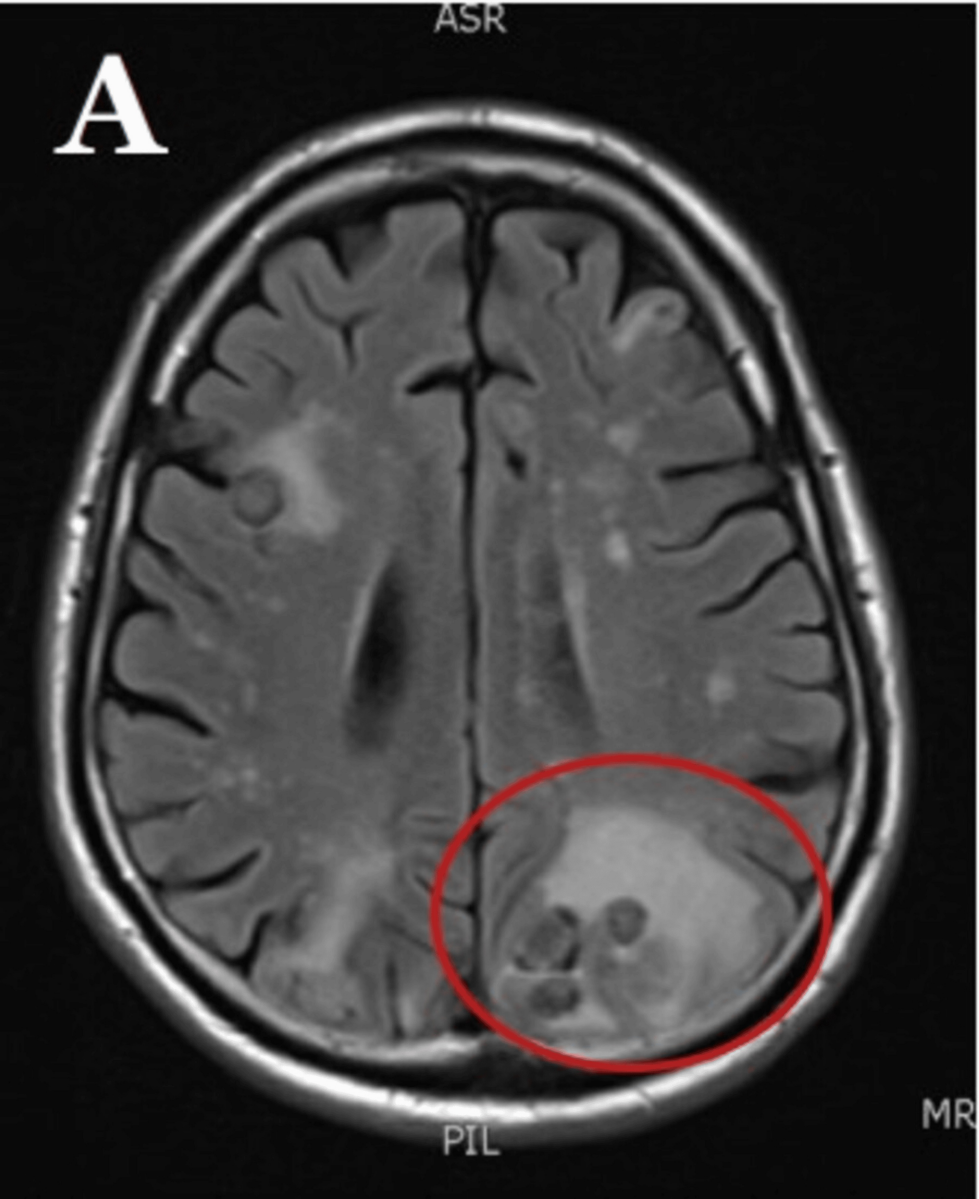
MRI brain demonstrating numerous enhancing brain lesions bilaterally, with the largest lesions including a 2.5 x 2.2 cm lesion in the left posterior temporal lobe

**Figure 2:**
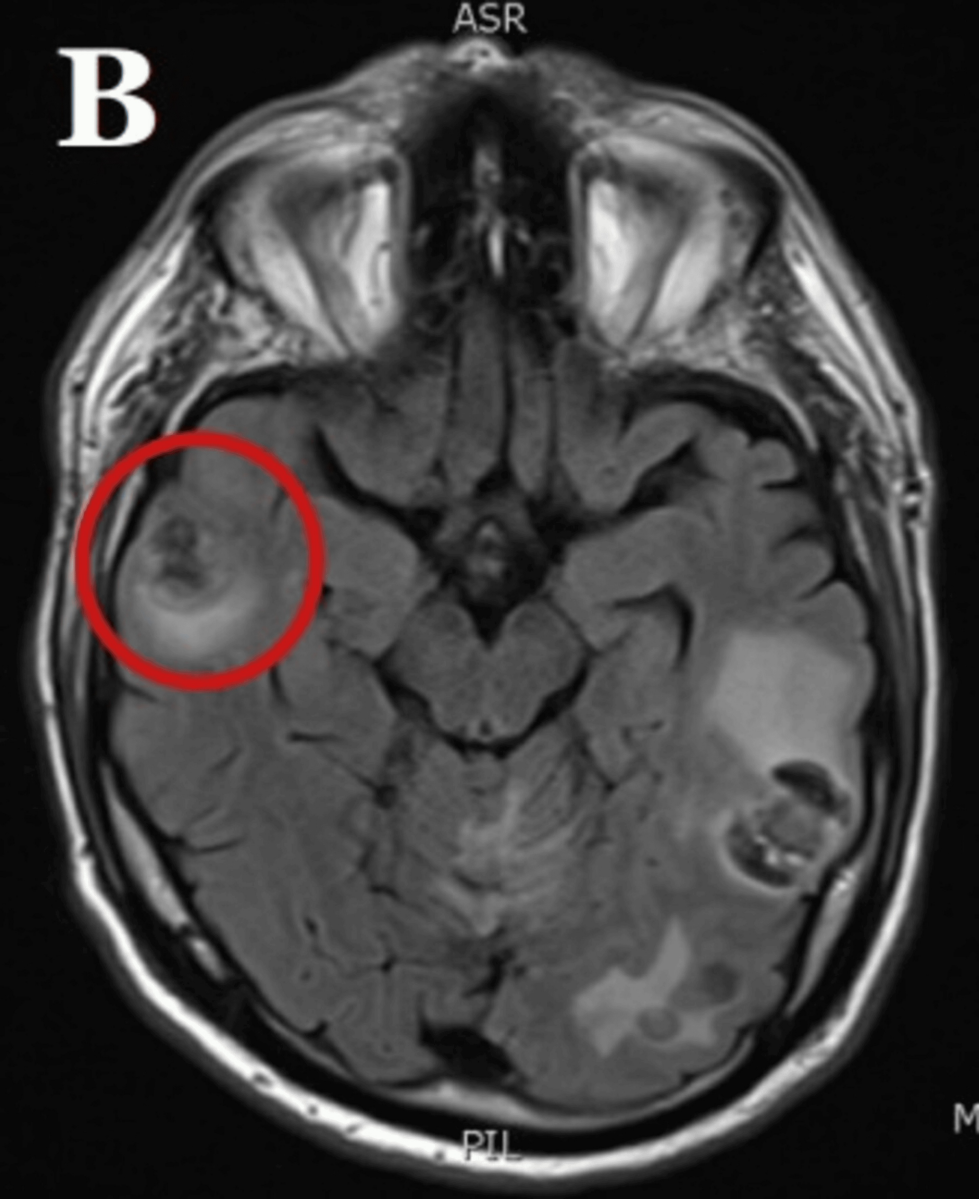
MRI brain showing a 1.4 x 1.0 cm lesion in the right lateral ventricle frontal horn and associated vasogenic edema surrounding the lesions

After appropriate treatment in the intensive care unit, the patient was transferred to the general floor but had persistent encephalopathy. On physical exam, he did not have any focal neurological deficits. However, he did demonstrate expressive aphasia, and his wife commented that he was not at his cognitive baseline. A computed tomography (CT) scan of the head was obtained, which showed multiple lesions with vasogenic edema concerning for metastatic cancer of unknown etiology. An electroencephalogram revealed moderately severe generalized cerebral dysfunction but no seizure activity. Magnetic resonance imaging (MRI) of the brain showed numerous bilateral enhancing lesions with surrounding vasogenic edema. The most significant was a 2.5 x 2.2 cm lesion in the left posterior temporal lobe and a lesion in the left cerebellum measuring 2.2 x 3.4 cm (Figure 1, Figure 2).

**Figure 3:**
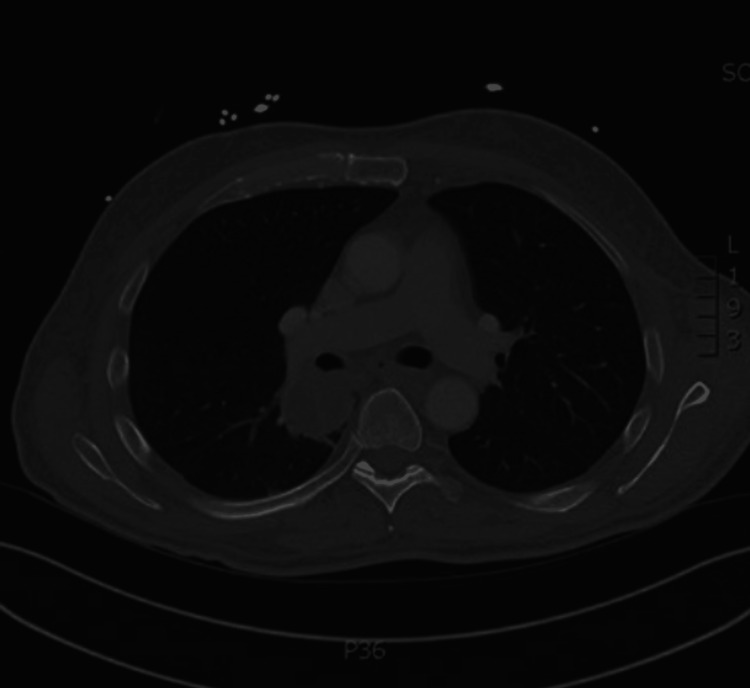
A computed tomography scan of the chest showing a large, heterogenous, predominantly right lower lobe mass measuring up to 4.3 cm with right mainstem bronchus/right lower lobe bronchus posterior wall abutment without significant luminal stenosis

Oncology and Pulmonology were consulted. Further workup involved CT scans of the chest, abdomen, and pelvis, which revealed a 4.3 cm heterogeneous lesion of the right lower lobe of the lung involving the mainstem bronchus without compression (Figure 3). Multiple sub-6 mm pulmonary nodules were also noted throughout (Figure 3). Dexamethasone was administered to the patient due to the vasogenic edema, and the next day, the patient's expressive aphasia had resolved [3].

Retrospectively, it was learned that the patient had initially presented with melanoma of his left shoulder (V600E mutation positive) and was treated with surgical resection. Sentinel biopsy revealed a positive lymph node at that time. Interferon therapy was attempted but discontinued due to poor tolerance. The patient was lost to follow-up for six years before presenting as above. At that time, his last positron emission tomography (PET)/CT and MRI were negative for distant disease.

Ultimately, this patient underwent a transbronchial biopsy of his right lower lobe mass, which confirmed metastatic melanoma. Hematology-Oncology decided to initiate whole-brain radiation inpatient, and he was discharged the following week.

Our patient presented a month after discharge with worsening leg weakness and intermittent confusion. CT revealed progression of his brain metastasis, causing mass effect at the right frontal horn, an L-to-R midline shift of the fourth ventricle, and an R-to-L midline shift. On physical examination, he was hard of hearing and had continued expressive aphasia and mild cognitive difficulty. The patient's expressive aphasia likely resulted from the 2.5 x 2.2 cm left posterior temporal lobe lesion, affecting Broca's or Wernicke's areas. Resolution of aphasia following dexamethasone suggests edema-related dysfunction rather than permanent neuronal loss. Neurosurgery was consulted, but surgical intervention was not pursued given his extensive tumor burden. Instead, our patient underwent a second steroid taper and was discharged to his oncologist to pursue a trial of disease-modifying treatment with dabrafenib/trametinib [4].

## Discussion

On average, the median time from primary melanoma diagnosis to metastasis is 3.2 years [1]. This patient presented with metastasis to the lung and brain nine years after the initial diagnosis of primary melanoma, and his PET/CT and MRI three years after the primary diagnosis were negative for distant disease.

Treatment for metastatic melanoma is determined by the number of metastases, location, and size of metastases, prior treatment for melanoma, presence of a BRAF mutation, presence of extracranial metastases, and the patient’s current performance status [2]. Surgery is potentially curative but generally reserved for patients with fewer than three metastases. Surgery is typically reserved for patients with one to three metastases in non-eloquent brain areas, though decisions depend on tumor size, symptoms, and patient performance status [5].

Local control is a high priority for patients with symptomatic brain metastasis or multiple large brain metastases. These patients often require a course of glucocorticoids to manage central nervous system (CNS) symptoms in conjunction with definitive locoregional CNS therapy. In patients who harbor BRAF-mutant bulky extracranial metastases, such as our patient, targeted therapy with BRAF plus MEK inhibitors can often provide a rapid radiographic and clinical response. Intracranial responses are generally of shorter duration than extracranial responses [6].

Historically, single-agent BRAF inhibitors were used until the landmark COMBI-AD trial. This trial showed that compared to a placebo, a combination of dabrafenib (BRAF inhibitor) and trametinib (MEK inhibitor) improved relapse-free survival at three years from 29% to 58% [7]. This study did not include patients with metastasis to the brain, as our patient had; however, he was treated with a combination of dabrafenib and trametinib as suggested by the small open-labeled cohort study COMBI-MB. In this trial, a radiographic response was seen in 50% of patients with melanoma-confirmed brain metastasis. Also, the median time to death was seven months compared to three months in prior studies when whole-brain radiation was the standard of care [8,9].

The presentation of metastatic melanoma in the brain varies based on location and size. Patients may experience headaches, fatigue, vomiting, nausea, unsteadiness, weakness, slurred speech, facial droop, or even seizures [9,10]. This patient’s most unusual symptom included aphasia, initially thought to be due to his DKA episode. Looking at the predominant location of his metastases (temporal and frontal), these sites seem to correlate with his deficit and even match his repeat presentation with his difficulty in hearing and processing auditory information.

## Conclusions

Brain metastases develop in nearly half the patients with advanced melanoma. In patients with a history of melanoma presenting with encephalopathy or neurological symptoms, suspicion of a metastatic brain mass should remain high, especially if altered mental status or neurological deficits are present. The most common symptoms of brain metastasis consist of headaches, weakness, seizures, vision changes, and nausea or vomiting. This case underscores that brain metastases occur in ~50% of advanced melanoma patients, often with delayed onset. While metabolic etiologies (e.g., DKA) may cloud initial assessment, focal deficits (aphasia) or persistent encephalopathy mandate CNS imaging. Symptoms reflect lesion locations, and targeted therapies (e.g., BRAF/MEK inhibitors) can mitigate progression, as demonstrated here.
